# Quality of Life for Saudi Patients With Heart Failure: A Cross-Sectional Correlational Study

**DOI:** 10.5539/gjhs.v8n3p49

**Published:** 2015-06-25

**Authors:** Mohannad Eid AbuRuz, Fawwaz Alaloul, Ahmed Saifan, Rami Masa’Deh, Said Abusalem

**Affiliations:** 1 College of Nursing, Applied Science University, Amman, Jordan; 2 King Fahad Specialist Hospital, Dammam, KSA; 3 School of Nursing, University of Louisville, Louisville, Kentucky, USA

**Keywords:** Arab world, ejection fraction, GCC, heart failure, Medical Outcomes Study-Social Support Survey, Middle East, quality of life, Saudi Arabia, Short Form-36, social support

## Abstract

**Introduction:**

Heart failure is a major public health issue and a growing concern in developing countries, including Saudi Arabia. Most related research was conducted in Western cultures and may have limited applicability for individuals in Saudi Arabia. Thus, this study assesses the quality of life of Saudi patients with heart failure.

**Materials and Methods:**

A cross-sectional correlational design was used on a convenient sample of 103 patients with heart failure. Data were collected using the Short Form-36 and the Medical Outcomes Study-Social Support Survey.

**Results:**

Overall, the patients’ scores were low for all domains of Quality of Life. The Physical Component Summary and Mental Component Summary mean scores and SDs were (36.7±12.4, 48.8±6.5) respectively, indicating poor Quality of Life. Left ventricular ejection fraction was the strongest predictor of both physical and mental summaries.

**Conclusion:**

Identifying factors that impact quality of life for Saudi heart failure patients is important in identifying and meeting their physical and psychosocial needs.

## 1. Introduction

Heart failure (HF) is a chronic syndrome characterized by significant physical, psychological and social burdens, resulting in poor quality of life (QoL) (Demir & Unsar, 2011). It is a chronic and progressive clinical syndrome associated with increased neuro-hormonal activity and multiple organ dysfunction (Fotos et al., 2013). HF is a major public health issue, with 23 million cases worldwide, and rising (Liu & Eisen, 2014). Projections show that by 2030 the prevalence of HF will increase by 25% from 2013 estimates in the US (Heidenreich et al., 2011). The American Heart Association (AHA) estimates that 5.7 million Americans older than 20 years have HF based upon 2007-2010 data (Roger et al., 2012), representing an annual cost of $34.4 billion annually (Heidenreich et al., 2011). The incidence of HF ranges between 1% and 19.3 per 1,000 person-years in older adults ≥65 years (Bui, Horwich, & Fonarow, 2011; Roger et al., 2012).

Approximately 6.5 million patients in Europe and 2.5 million patients in Japan suffer from HF (Fotos et al., 2013). In the UK, HF affects an estimated 3% of people aged between 65 and 74 years; this prevalence increases with age and affects approximately 15% of the population aged over 74 (Riley, 2014). Similar to the global picture, HF is also a growing concern in developing countries (Pelegrino, Dantas, & Clark, 2011). The extrapolated prevalence of HF in Saudi Arabia is 455,222 cases and the estimated incidence is 32,200 cases annually (Health Grades Inc., 2012). A recent study by Albackr et al. (2013) showed that 20% of the patients who were admitted to Saudi hospitals with acute coronary syndrome (ACS) had HF.

HF has negative impacts on all aspects of patients’ physical, social, psychological, emotional and spiritual well-being (Heo et al., 2009; Chen et al., 2010). HF patients have difficulties in performing activities of daily living; suffer from economic, sexual and psychosocial problems; and encounter troubles in social and professional relationships (Demir & Unsar, 2011; Pelegrino et al., 2011). These negative consequences are frequently due to secondary symptoms associated with HF such as fatigue, shortness of breath, insomnia, drowsiness and anxiety (Heo et al., 2008, 2009). Therefore, nursing interventions to improve QoL for patients with HF is warranted. The objectives of HF treatment are to maximize life expectancy, improve QoL, and prevent disease progression and hospital admissions.

In order to identify effective interventions to improve QoL, it is imperative to identify factors associated with changes in QoL among patients with HF, including age, gender, social support and marital status (Pelegrino et al., 2011), functional class, duration of HF, left ventricular ejection fraction (LVEF), other comorbidities and psychosocial status (Chung et al., 2012; Son et al., 2012).

Culture plays an important role in defining health, sickness, and shapes individuals’ QoL (Padilla, Kagawa-Singer, & Ashing-Giwa, 2012). Therefore, the World Health Organization (WHO, 1995) emphasized the role of culture in shaping QoL and defined it as the “Individual's perception of his/her position in life in the context of the culture and value systems in which he/she lives, and in relation to his/her goals, expectations, standards and concerns” (p.1405). Huang et al. (2010) found that QoL among Taiwanese patients was better than among American patients with HF due to cultural differences.

There are differences between Arab-Islamic and Western cultures in terms of customs, relationships and values. Saudi Arabia is a Middle Eastern, Arabic, Islamic country characterized by a collectivist society with strong family and community ties, abiding Islamic faith, restraint in disclosing personal matters, including health-related issues (Wehbe-Alamah, 2008), and a male-dominated culture (Al-Krenawi & Graham, 2000). Arab patients may prefer not to express their physical and mental complaints that consequently impact their health and QoL. They may rely on their faith in relieving their discomfort. Hence, outcomes of research conducted in Western culture may be or may be not applicable to individuals residing in Saudi Arabia. Therefore, this study was conducted to determine if research outcomes from Western culture can be applied to Saudi Arabian patients with HF.

Social support has positive outcomes on different aspects of patients’ health. Social support was found to reduce mortality, enhance healthy lifestyles, provide a buffer against adverse life events (Staniute, Brozaitiene, & Bunevicius, 2013) and improve QoL (Sammarco & Konecny, 2010; Wang, Lau, Chow, Thompson, & He, 2014). Muslim and Arabic cultures are known to provide social support to the individuals when they are sick or in need for help. However, the association of perceived social support with QoL among Saudi Arab and Muslim patients with HF has not been explored. So far, QoL for patients with HF has been studied mostly in the US (Heo et al., 2009; Pelegrino et al., 2011), Canada (Ducharme et al., 2005) and Europe (Fotos et al., 2013).

### 1.1 Aim

Given the massive negative effects of HF and its progressive nature, in addition to the lack of knowledge about QoL of HF patients in Saudi Arab patients, the purposes of this study were to: 1) identify demographic characteristics (age, sex, income, and marital status) and medical variables (LVEF, HF duration, presence of diabetes mellitus, and hypertension) associated with QoL; (2) examine the relationships of social support dimensions with QoL in patients with HF; and (3) identify variables that explain the greatest amount of variance in physical component summary (PCS) and mental component summary (MCS) of QoL.

## 2. Methods

### 2.1 Design, Sample and Setting

A cross-sectional correlational design was used. A convenient sample of 103 patients with HF was adequate to detect statistical significant results based on 80% power, a Cronbach's alpha of 0.05, 12 independent variables, and an estimated effect size of 0.2 (Soper, 2013). The inclusion criteria were patients diagnosed with HF able to read and write in Arabic. Patients were excluded from the study if they had cancer or major psychiatric problems that could affect the completion of the questionnaires or their QoL. The sample was recruited from a tertiary care hospital in the Eastern province of the Kingdom of Saudi Arabia. This tertiary care hospital is the largest hospital in the Eastern province, with a capacity of 1500 beds. Moreover, it is specialized in cardiac and oncology care, and receives referral cases from all other hospitals in the Eastern province, which has a population of approximately seven million. All other hospitals in that area are smaller hospitals. Based on this information, the patients included in this study were from all the cities of the Eastern province.

### 2.2 Measurement of Variables

QoL was measured using the Short Form-36 version 2 (SF36). This generic instrument to measure QoL has been widely used for patients with HF (Coelho et al., 2005). This tool is a 36-item multiple-response option questionnaire with eight scales: physical functioning (10 items), role physical functioning (four items), role emotional functioning (three items), vitality (four items), mental health (five items), social functioning (two items), body pain (two items), general health (five items), and one item comparing current patient's health with the last year (Ware & Sherbourne, 1992). Scales are measured on a 0-100 score. Higher scores indicate higher levels of QoL in each domain. Two major summaries can be calculated: Physical Component Summary (PCS), which consists of physical functioning, role physical functioning, body pain, general health, and Mental Component Summary (MCS), which consists of: role emotional functioning, vitality, mental health, and social functioning.

The psychometric properties of this instrument were evaluated for 1,980 patients aged16-74 years. The results showed that the instrument had satisfactory to excellent alpha reliability coefficients (Brazier et al., 1992), with Cronbach's alpha greater than 0.75 for all scales except social functioning (α = 0.73) (Brazier et al., 1992). The SF-36 also has an evidence of construct validity by a strong correlation with Nottingham Health Profile (Brazier et al., 1992). This instrument was translated into Arabic and has shown satisfactory psychometric results (Coons et al., 1998). The minimum Cronbach's alpha for general health was 0.71, and the highest was for physical functioning (0.94).

*Medical Outcomes Study-Social Support Survey (MOS-SSS)*: To assess perceived functional support, the MOS-SSS was used. The MOS-SSS survey consists of 19 self-reported questions (Sherbourne & Stewart, 1991). The first 18 items are divided into four subscales: emotional/informational support (eight items), affectionate support (three items), tangible support (four items) and positive social interaction (three items). These subscales have a five-point Likert scale ranging from 1 (none of the time) to 5 (all of the time). All raw scale scores are standardized by transforming them into a 0-100 scale. Item number 19 measures the availability of people to support respondents and keep their minds at peace. Higher scores for the subscales and for the overall support index indicate a higher level of support (Sherbourne & Stewart, 1991).

The internal consistency reliability for this instrument was evaluated using a sample of 2,987 patients with chronic conditions. The scale with lowest alpha score was positive social interaction (α = 0.91) and the highest alpha score scale was emotional/informational support scale (α = 0.96). The alpha for the overall support index was 0.97 (Sherbourne & Stewart, 1991). This questionnaire had strong convergent and discriminate validity. This instrument was translated into Arabic and had good internal consistency in a sample of 63 Arab stem cell transplant survivors. Cronbach's alphas ranged from 0.79 to 0.87(Alaloul, 2007).

*Demographic and medical characteristics*: A demographic form was developed by the researchers and by trained research assistants to collect data on participants’ age, sex, marital status, educational level, employment status and annual income. Additionally, medical variables including time since diagnosis with HF, LVEF, history of diabetes, or hypertension were included in the demographic form.

### 2.3 Procedure

Institutional review board (IRB) approval was obtained from King Fahd Specialist Hospital (Dammam) IRB Committee. The principal investigator met with administrators, physicians and the nursing director and explained the purposes and nature of the study. HF patients who met the inclusion criteria were contacted during their visit to the outpatient clinic. The study was explained and informed consent was obtained from patients who agreed to participate. Participants completed the questionnaire in a quiet, private place in the hospital. Each participant completed the SF-36, the MOS-SSS and the demographic data form. Two trained nurse research assistants obtained patients’ demographic characteristics and medical variable data from patients’ medical records.

### 2.4 Data Analysis

Data were analyzed using the Statistical Package for the Social Sciences for Windows 21.0 (SPSS, Inc., Chicago, Illinois). An alpha of < 0.05 was used to determine the statistical significance of analyses. Descriptive statistics (mean, standard deviation or frequency, and range) were used to assess participant's demographic and medical characteristics, the SF-36, and the MOS-SSS scores.

To identify factors associated with QoL, a series of bivariate correlations were conducted to test for associations between the two summary subscales of the SF-36 (physical and mental) and demographic variables (i.e., age, educational level, yearly income, marital status, and employment status), medical variables (LVEF, duration of HF, and the presence of hypertension or diabetes). Pearson r for interval measures, the Spearman ρ for ordinal measures, and the χ^2^ test for categorical measures. Predictor variables with significant correlation (p-value <0.1) were selected for further analysis. Next, stepwise regression analysis was used to evaluate the strength of association of demographic characteristics, medical variables, and perceived social support with two summary subscales of the SF-36 (physical and mental). For multivariate analyses, multicollinearity was assessed using bivariate correlations among predictor variables, tolerance indices, and variance inflation factors; no serious multicollinearity was found among predictor variables.

## 3. Results

### 3.1 Descriptive Statistics

The mean age of the participants was 50.3 years (SD = 16.3, rang: 19-90). The duration patients’ had HF ranged from 1-15 years, with a mean of 5.4 years. Their mean ejection fraction was 37.9% (SD = 5.7, range: 30-55). Approximately three-quarters (73%) of the sample had hypertension, and half had diabetes. Other participants’ demographic characteristics are presented in Table 1. Medical Outcomes Study-Social Support Survey Scores are presented in Table 2. The emotional/informational support dimension had the lowest mean score and tangible support had the highest mean score. The mean scores for the QoL domains, the PCS and the MCS are presented in Table 3. Overall, the patients’ scores were low for all domains indicating poor QoL. The PCS and MCS mean results were low, indicating poor QoL as well. The lowest mean score was for General Health (General perception about health), which meant that the patients felt poor QoL, whereas mental health had the highest mean score.

**Table 1:** Demographic Characteristics of Patients with Heart Failure (*N* = 103)

Characteristics	n (%)
**Gender**	
Male	60 (58.3)
Female	43 (41.7)
**Marital status**	
Single	15(14.5)
Married	63 (61.2)
Divorced	8 (7.8)
Widowed	17 (16.5)
**Employment**	
Employed	74 (71.9)
**Educational level**	
Less than high school/High School	18 (17.5)
Diploma	24 (23.3)
Holding Bsc	61 (59.2)
**Income**	
Less than 3000 SR	29 (28.2)
From 3001-5000SR	18 (17.5)
5001-10000SR	31 (30.1)
More than 10000 SR	25 (24.3)

SR: Saudi Riyals.

**Table 2:** Medical Outcomes Study-Social Support Survey Scores of the Saudi Patients with Heart Failure (*N* = 103)

Scale/Subscale	Mean	SD	Possible Range	Actual Range
Emotional/Information Support	60.6	13.9	0-100	25-100
Tangible Support	84.7	12.1	0-100	25-100
Affectionate Support	74.0	12.7	0-100	25-75
Positive Social Interaction	63.8	12.5	0-100	25-75
Total score	65.6	12.7	0-100	25-80

**Table 3:** Scores for the SF-36 Quality of Life Domains, PCS, and MCS of the Participants (*N* = 103)

Subscale	Mean	SD	Possible Range	Actual Range
Physical Functioning (Level of limitation in physical activities)	42.9	14.1	0-100	0-80.0
Role Physical (Problems with usual role related to physical health)	43.2	11.5	0-100	25-75.0
Social Functioning (Interference with social activities)	45.0	10.7	0-100	12.5-62.5
Role Emotional (Problems with usual role related to emotional health)	45.2	9.6	0-100	25.0-75.0
Bodily Pain (Level of pain)	33.9	11.5	0-100	22.5-67.5
General Health (General perception about health)	26.8	12.5	0-100	0-55.0
Vitality (Energy and fatigue)	51.6	6.8	0-100	31.3-68.8
Mental Health (Level of psychological status)	53.4	8.3	0-100	35.0-75.0
*Physical component summary*	*36.7*	*12.4*	*0-100*	*13.3-61.6*
*Mental Component summary*	*48.8*	*6.5*	*0-100*	*30.3-63.0*

Age, ejection fraction, and tangible support were significantly correlated with both PCS and MCS (Table 4). Therefore, these variables were included in stepwise regression. These three variables explained 39% of total PCS subscale variation. Age, and LVEF explained 27% of total MCS subscale variation (Table 5).

**Table 4:** Correlations between physical and mental component summaries and Selected Significant Demographic, Medical Variables and Social Support Subscales (N = 103)

Variables	Age	LVEF	History of HTN	History of DM	Emotional Support	Tangible Support	Affectionate Support	Positive Social Interaction
Physical component summary	-.457**	.613**	-.352**	-.201*	.360**	.454**	.319**	.386**
Mental component summary	.516**	.451**	-.320**	-.238*	.469**	.324**	NS	.343**

LVEF: Left ventricular ejection fraction; HTN: hypertension; DM: Diabetes Mellitus;

** significant, P<.001;

* significant at P<.05; NS: Not significant.

**Table 5:** Regression Analyses: Predictors of the PCS and MCS of the SF-36 (N = 103)

Outcomes/Predictors	Standardized β	t	Model Statistics
Physical component summary			
Ejection Fraction	0.61	7.8	*R*^2^ = 0.39; *F(3,99)*= 60.7, *p* <. 001
Tangible Support	0.29	3.8	
Age	-0.25	-3.4	
Mental component summary			
Ejection Fraction	0.37	3.8
Age	-0.21	-4.9

## 4. Discussion

In this study, Saudi patients reported poor QoL in all domains, PCS, and MCS of the SF36. Moreover, the general perceptions of health and pain domains had the lowest scores respectively, which indicate that they know that their heath is poor and affecting their QoL. We drew this conclusion when we compared the results of this study with the mean norms SF-36v2 of Maglinte, Hays, and Kaplan (2012), which was conducted in a developed country. Inconsistent with our findings, Peters-Klimm et al. (2010) found that social functioning and role emotional were slightly impaired. In Turkey, which has similar cultural characteristics, similar findings were found (Demir & Unsar, 2011)

Culture, knowledge, attitudes, beliefs or health care system issues are potential explanations for these differences (Huang et al., 2010). Saudis prefer not to disclose their emotions during illness to the public and may avoid seeking emotional and social help from healthcare providers (Al-Busaidi, 2010), although they ask for assistance to meet their physical needs, which are impossible to hide. Patients received tangible support from their first-degree family members and the personal attendants who are always around sick people in Saudi Arabia for socio-cultural and religious reasons. Our findings showed that tangible support has the highest mean among all support subscales. Further studies are paramount to explore differences in QoL among different cultures.

The findings of this study are comparable to those of Hoekstra et al. (2011) who found that mental health QoL domain was slightly compromised among patients with HF from the Netherlands. In our study, mental health domain had the highest score (53.4, SD= 8.3), which is still slightly below the mean found by Maglinte et al. (2012) of 54.27 (SD = 13.28), which indicates that it is slightly compromised. Maglinte et al. (2012) found that the mental component domain was the highest among all domains consistent with our study findings. We found that the mental health domain has the highest score that contributes to the improvement of QoL; however, it was not statistically significant in the regression models. Also, consistent with our study, several studies reported impaired pain domain scores (Rustoen et al., 2008; Nesvold et al., 2011). Different reasons need to make the health care providers pay more attention to pain assessment and management among Saudi patients. For Saudi participating patients, pain is part of their chronic illness and may be seen as a way to purify (expiate) their sins (Al-Busaidi, 2010; Van den Branden, 2010).

When examining the relationship between demographic and medical variables associated with QoL in Saudi patients with HF, LVEF had the strongest correlation with QoL among Saudi patients followed by age. These two variables were significant in predicting all QoL domains. Other variables that have a significant relation with the QoL were the Tangible Support, history of HTN, and DM. However, only Tangible Support remained a significant predictor of QoL.

Low LVEF, which is an objective clinical measurement of HF, leads to pulmonary edema resulting in fatigue and dyspnea. Different studies showed that LVEF is a strong predictor of mortality (Rizzello et al., 2009; Banovic et al., 2011) and hospital admissions (Chaudhry et al., 2013) for patients with HF. Two studies (Heo et al., 2008; Pelegrino et al., 2011) showed that there is a relationship between LVEF and QoL. They also found that there is a significant positive correlation between LVEF and QoL. In this study, high levels of LVEF were associated with better QoL. Therefore, effective management of LVEF might improve patients’ overall signs and symptoms, reduce mortality rate, hospitalization, fatigue and enhanced QoL.

The results of this study showed that younger people had better PCS and MCS scores, which are associated with better QoL. Consistent with previous studies (Fotos et al., 2013; Iqbal et al., 2010; Lewis et al., 2007), younger age was significantly correlated with higher PCS score, higher physical functioningand higher role physical domain scores of the SF-36. On the other hand, being older was associated with better mental component and mental health domain SF-36 scores.

Heo et al. (2008) found that age independently predicted QoL and physical symptoms at one and three months’ follow-up; younger patients had better QoL and better PCS activities. Younger patients reported performing more activities of daily living and had better QoL in a study in Turkey (Demir & Unsar, 2011). Age remained significant in all regression models for all domains of QoL when it was checked among overweight or obese adults (Wang et al., 2013). When Pelegrino et al. (2011) checked health-related QoL determinants in outpatients with HF, they found a negative significant relationship between age and QoL. Patients aged ≥ 65 years had poorer QoL compared to those aged < 65 years, which is consistent with our study findings.

One of our aims in this study was to describe the association of perceived social support with QoL among participating patients with HF. Patients with HF received a moderate to high level of social support. Previous studies (Bennett et al., 2001; Barutcu & Mert, 2013) showed nearly the same results. Barutcu & Mert (2013) found that Turkish HF patients, who are mostly Sunni Muslims (and thus sharing many socio-cultural traits with patients in Saudi Arabia), received extensive social support from their families (Tangible Support). Tangible Support had the highest scores among all types of support and remained significant in all regression models in both MCS and PCS. This is not surprising since patients with these compromised summaries due to the disease process and treatment complexity were more in need of tangible support (Tajvar et al., 2013).

Moreover, all support subscales had a positive correlation with the PCS and MCS subscales indicting that this support will improve the QoL for HF patients, including participating patients in this study. Fotos et al. (2013) found that unmarried patients with HF have poorer QoL than married patients, which supports the relationship of the Tangible Support provided by the family members and other people with the improvement of QoL. Therefore, establishing support groups and encouraging patients to seek support from friends, the community, and healthcare providers in addition to their families might improve their perceived social support in all dimensions.

## 5. Conclusion

Participating Saudi patients with HF reported poor QoL in all domains, PCS and MCS. They had moderate to high support scores indicating a positive affect on their QoL but their general QoL scores were still low. Older adult patients with acutely low LVEF had the lowest QoL scores, indicating a need for further attention and management. The holistic care for patients with HF by a multidisciplinary team of healthcare professionals and families could improve their QoL.
